# Clinical effectiveness/child-patient and parent satisfaction of two topical fluoride treatments for caries: a randomised clinical trial

**DOI:** 10.1038/s41598-024-58850-w

**Published:** 2024-04-07

**Authors:** Ilze Maldupa, Nicola Innes, Ilona Viduskalne, Anda Brinkmane, Egita Senakola, Karina Krumina, Sergio E. Uribe

**Affiliations:** 1 Department of Conservative Dentistry and Oral Health, Riga Stradins University, Dzirciema str. 20, Riga, 1007 Latvia; 2 School of Dentistry, College of Biomedical and Life Sciences, Cardiff University, Cardiff, Wales, UK; 3 Baltic Biomaterials Centre of Excellence, Headquarters at Riga Technical University, Riga, Latvia; 4 Faculty of Dentistry, Universidad de Valparaíso, Valparaíso, Chile

**Keywords:** Silver diammine fluoride, Non-invasive caries management, Early childhood caries, Randomised controlled trial, Patient-centred outcomes research, Diseases, Health care, Medical research

## Abstract

Knowledge gaps exist regarding optimal silver diammine fluoride (SDF) regimens and the efficacy of new products for arresting dental caries in young children. We evaluated the effectiveness of 38%-SDF (SDI-RivaStar), Tiefenfluorid (TF) comparing with Placebo (P), all in conjunction with behavioural modification (BM), in preventing major complications (endodontic/extractions/pain)—a patient-centred outcome—due to early childhood caries over 12 months in children under 71-months. A six-arm, patient/parent-blinded, superiority, placebo-controlled randomised control trial at the university clinic in Riga, Latvia, from 1/9/20-31/8/22 (Protocol registration ISRCTN17005348). The trial tested six protocols, using three compounds (P/SDF/TF) under two regimes: annual and biannual (P1/P2/TF1/TF2/SDF1/SDF2) for major complications. Secondary outcomes included minor complications and parental satisfaction. All groups received BM. 373/427 randomised children (87.3%) completed the study. SDF2 had a significantly lower rate and risk of major (21.5%, OR = 0.28, 95%CI [0.11, 0.72], *p* < 0.05) and minor complications (OR = 0.16 (95%CI [0.05, 0.50], *p* = 0.002). Overall satisfaction was 96% (*p* > 0.05). SDF biannual application with BM effectively prevented major complications of early childhood caries and was well accepted by children and their parents. Trial registration number: ISRCTN17005348, principal investigator: Ilze Maldupa, registration date: 30/06/2021.

Clinical trial registration number: ISRCTN registry: ISRCTN17005348, https://doi.org/10.1186/ISRCTN17005348, registration date: 30/06/2021.

## Introduction

Oral health is an essential element of general health and positively influences child development^1^. Caries develops as a result of behaviours, and complex evidence-based interventions are needed to prevent or manage it and halt further progress of the disease^2^. Non-invasive caries treatment strategies have been proposed. In addition to being non-invasive, they have the additional advantage of lowering care costs and decreasing the number of tooth extractions^3^. Notably, silver diammine fluoride (SDF) has been found to have up to 89% higher efficacy than other treatments or placebos^4,5^. However its use is limited by its poor aesthetic appearance. The optimal application frequency for SDF treatment remains unresolved, and there is a lack of research on the comparative effectiveness of different products^6^ Also, new formulations, such as Tiefenfluorid™ (TF), have gained significant traction in some countries such as Latvia, for their ability to remineralise teeth without the drawback of black staining, as demonstrated by in vitro studies^7,8^. However, clinical trials comparing existing and new formulations, are lacking.

Dental caries significantly affects children, causing pain, difficulty sleeping, loss of time from school and is expensive to treat^9^; however, research in dentistry has traditionally prioritised clinician-centred measures, such as arresting dental caries or preventing lesion progression^4,11^ expressed numerically and neglecting patient-centred outcomes (PCOs)^12^.

While it is widely acknowledged that childhood caries is linked to family behaviour^13^ and that behaviour change is a complex process^14^, family motivation is still seldom taken into account in clinical trials^15^. To promote the health and well-being of children beyond mechanical or restorative treatments, it is crucial to prioritise research that can help transfer evidence-based practices into real-world settings^16^. For scaling-up evidence-based interventions in national health systems^2^ for the treatment of early childhood caries, there needs to be a more comprehensive understanding of fluoride compounds and their optimal use, combined with behaviour change interventions. These must be understood within the context of the outcome measures expressed in PCOs.

This placebo-controlled randomised clinical trial, aimed to evaluate the effectiveness of two fluoride-containing products (SDF and Tiefenfluorid) and a placebo, using two different application protocols (annual/biannual) in children under 6 years of age. All groups, including the placebo, received a behavioural management (BM) intervention as part of the treatment protocol, making it a complex intervention. The outcomes were measured at 12 months. The focus was on effectiveness from a patient-centred perspective, defined through the primary outcome of the proportion of major complications (parents-declared dental pain, tooth extraction, or endodontic treatment in the last 12 months) and the secondary outcomes of effectiveness arresting carious lesions (minor complications), and parental and children’s satisfaction (through three outcomes: (1) would the parent be happy to have that treatment again for the same child or another of their children; (2) parent satisfaction with appearance and (3) child satisfaction with appearance). The outcomes, although broken down, were designed as composite variables to capture important treatment outcomes for parents and children and ensure that our findings were patient-centred.

## Results

### Baseline characteristics

**Figure 1:**
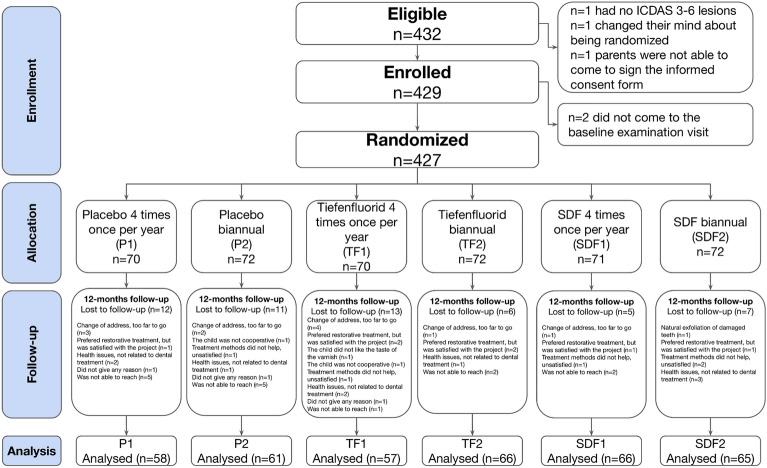
CONSORT Flow diagram illustrating participant enrollment, randomisation, allocation, follow-up, and analysis.

A total of 432 children were screened as eligible, and 427 enrolled, randomised, received treatment and were included in the analyses. Each group comprised 70–72 children (Fig. 1) at the start of treatment and 57–66 at the final 12-month analysis, giving 373 participants examined at the 12-month follow-up, a 13% attrition rate. The percentage of missing data was less than 5%, and missing values were found to be missing at random based on the MCAR (Little’s Missing Completely at Random) test. See Table 1 for further details on the composition of the study groups. Initially, imbalances were visually detected between groups regarding toothbrushing frequency, d1mft, d3mft, and active lesions, which could have caused a chance bias. To address this, we incorporated these variables into the linear adjustment using a regression model to mitigate the potential for bias. The interventions were delivered as planned without personalisation or adaptation. Adherence to the intervention was over 70% in all groups (Supplementary material, Table S1).

**Table 1:** Baseline demographic characteristics and oral health-related behaviours of children in each study group.

	P1	P2	TF1	TF2	SDF1	SDF2	Overall
	(N = 70)	(N = 72)	(N = 70)	(N = 72)	(N = 71)	(N = 72)	(N = 427)
Age (months)							
Mean (SD)	48.0 (12.0)	46.3 (15.3)	45.3 (13.5)	45.3 (14.8)	44.8 (13.2)	44.7 (15.4)	45.7 (14.1)
Median [Min, Max]	49.0 [16.0, 71.0]	45.5 [5.00, 72.0]	44.0 [5.00, 72.0]	45.5 [6.00, 76.0]	44.0 [8.00, 69.0]	46.5 [7.00, 70.0]	46.0 [5.00, 76.0]
Sex							
Female	31 (44.3%)	35 (48.6%)	30 (42.9%)	28 (38.9%)	26 (36.6%)	28 (38.9%)	178 (41.7%)
Male	39 (55.7%)	37 (51.4%)	40 (57.1%)	44 (61.1%)	45 (63.4%)	44 (61.1%)	249 (58.3%)
Toothbrushing frequency							
Twice or more	23 (32.9%)	13 (18.1%)	13 (18.6%)	17 (23.6%)	15 (21.1%)	25 (34.7%)	106 (24.8%)
Every evening	29 (41.4%)	38 (52.8%)	35 (50.0%)	35 (48.6%)	39 (54.9%)	24 (33.3%)	200 (46.8%)
Less	18 (25.8%)	21 (29.2%)	22 (31.5%)	20 (27.8%)	17 (23.9%)	23 (31.9%)	121 (28.4%)
Toothpaste							
Fluoride free	0 (0%)	2 (2.8%)	1 (1.4%)	3 (4.2%)	3 (4.2%)	3 (4.2%)	12 (2.7%)
< 1000 ppm F	42 (60.0%)	45 (62.5%)	43 (61.4%)	35 (48.6%)	36 (50.7%)	40 (55.6%)	241 (56.3%)
1000–1500 ppm F	28 (40.0%)	25 (34.7%)	26 (37.1%)	34 (47.2%)	32 (45.1%)	29 (40.3%)	174 (40.7%)
Sweets daily							
Yes	59 (84.3%)	54 (75.0%)	63 (90.0%)	57 (79.2%)	63 (88.7%)	62 (86.1%)	358 (83.8%)
Sugary drinks daily							
Yes	47 (67.1%)	49 (68.1%)	47 (67.1%)	53 (73.6%)	54 (76.1%)	41 (56.9%)	291 (68.1%)
Visible plaque							
Yes	41 (58.6%)	51 (70.8%)	51 (72.9%)	35 (48.6%)	46 (64.8%)	51 (70.8%)	275 (64.4%)
d1mft^†^							
Mean (SD)	9.06 (4.73)	8.82 (4.99)	10.4 (4.94)	8.33 (4.29)	9.93 (4.31)	6.64 (4.59)	8.85 (4.78)
Median [Min, Max]	9.00 [1.00, 20.0]	8.00 [1.00, 20.0]	10.0 [1.00, 20.0]	8.00 [2.00, 20.0]	10.0 [2.00, 20.0]	6.00 [1.00, 20.0]	8.00 [1.00, 20.0]
d3mft^‡^							
Mean (SD)	6.46 (3.72)	6.85 (4.56)	7.60 (4.50)	5.68 (3.31)	7.55 (3.87)	5.36 (3.64)	6.58 (4.03)
Median [Min, Max]	6.00 [1.00, 18.0]	6.50 [1.00, 20.0]	6.00 [1.00, 20.0]	5.00 [1.00, 14.0]	7.00 [1.00, 18.0]	4.00 [1.00, 15.0]	6.00 [1.00, 20.0]
Active lesions at baseline							
Mean (SD)	7.33 (5.06)	6.56 (4.64)	9.17 (4.75)	6.40 (4.66)	8.72 (4.13)	5.60 (4.05)	7.28 (4.71)
Median [Min, Max]	6.00 [0, 20.0]	5.50 [0, 20.0]	8.50 [1.00, 20.0]	5.50 [0, 20.0]	8.00 [2.00, 20.0]	5.00 [0, 20.0]	6.00 [0, 20.0]

^†^d1mft = damaged (ICDAS = 1–6), missed and filled teeth.

^‡^d3mft = damaged (ICDAS = 3–6), missed and filled teeth.

### Primary outcome

**Figure 2:**
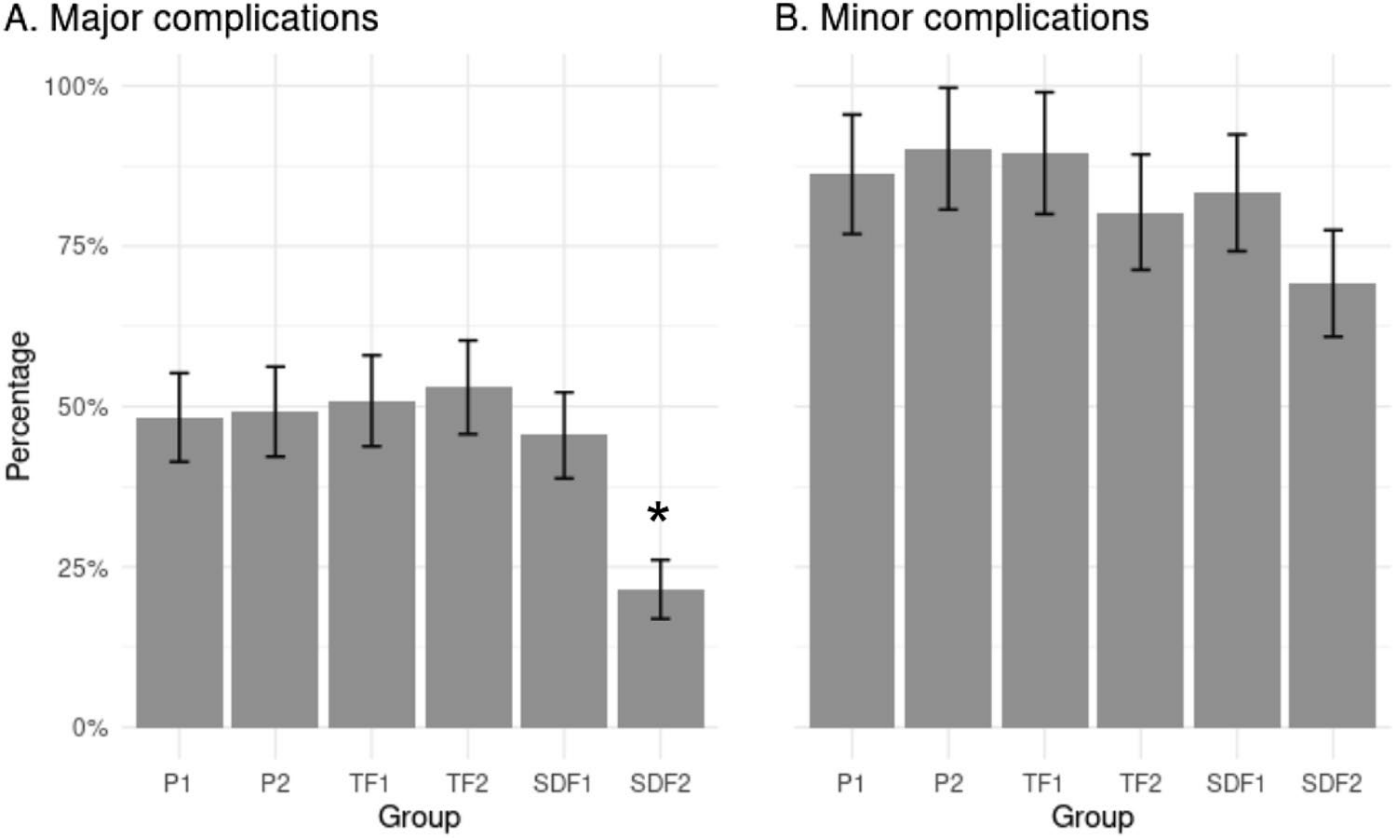
The proportion of (**A**) major and (**B**) minor complications by intervention group (percentage and standard deviation, * unadjusted proportions test  < 0.05).

The proportion (standard deviation) of major complications in each group were as follows: P1 = 48.3% (6.9), P2 = 49.2% (7.0), TF1 = 50.9% (7.1), TF2 = 53.0% (7.3), SDF1 = 45.5% (6.7) and SDF2 = 21.5% (4.6). The group SDF2 presented the lowest proportion of major complications, which was significant (X^2^ = 17.66, df = 5, *p*-value = 0.003) compared to P2. Figure 2A presents the results of the major and minor complications. The unadjusted analysis revealed a visual interaction between the compound and frequency of application, suggesting the effectiveness of SDF2 in reducing major complications (Supplementary material, Fig. 1); therefore, a General Lineal Model (GLM)-adjusted was used to confirm these findings. The adjusted GLM included various predictors and covariates for potential clustering, as shown in Table 2. The model’s baseline intercept for the P2 group was 3.16 (95%CI [1.61, 4.72], *p* < 0.001). The SDF2 intervention resulted in a significant reduction in the odds of major complications (OR = 0.28, 95%CI [0.11, 0.72]), while the other intervention groups did not show a significant difference compared with the placebo group after adjusting for covariates (Supplementary material, Table S2).

**Table 2:** Association between intervention groups and major and minor complications risk: odds ratios and 95%CI from the adjusted regression model.

	Major complications			Minor complications		
	OR^†^	95%CI^†^	*p*-value	OR^†^	95%CI^†^	*p*-value
Intervention						
P2—Placebo single applications, twice per year	–	–		–	–	
P1—Placebo one annual regime (four applications, one week apart)	1.08	0.46, 2.52	0.9	0.52	0.15, 1.84	0.3
SDF1—SDF one annual regime (four applications, one week apart)	0.74	0.33, 1.67	0.5	0.34	0.10, 1.19	0.091
SDF2—SDF single applications, twice per year	0.28	0.11, 0.72	0.008	0.17	0.05, 0.54	0.003
TF1—Tiefenfluorid one annual regime (four applications, one week apart)	0.94	0.40, 2.19	0.9	0.76	0.19, 3.01	0.7
TF2—Tiefenfluorid single applications, twice per year	1.96	0.86, 4.44	0.1	0.58	0.18, 1.88	0.4

^†^*OR* Odds Ratio, *CI* Confidence Interval.

### Secondary outcomes

For minor complications, there was no evidence of a difference in the proportion of minor complications (Fig. 2B) across the different intervention groups (X^2^ = 7.07, df = 5, *p* = 0.214). A mixed logistic model was used to predict minimal complications, with fixed effects for covariates and random effects for patients. The model’s marginal R^2^ related to fixed effects was 0.46. SDF2 significantly reduced the risk of minimal complications, with an odds ratio of 0.16 (95%CI [0.05, 0.50], *p* = 0.002). The effects of interventions P1, TF1, TF2, and SDF1 were not statistically significant. Details are presented in Table 2.

**Figure 3:**
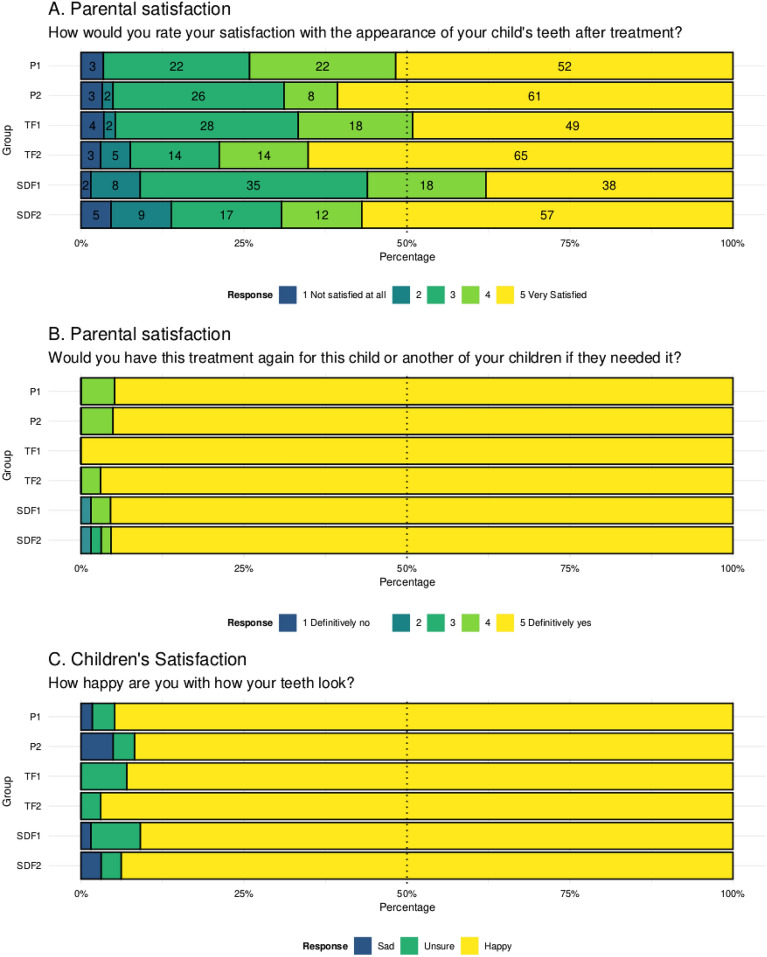
Parental and children’s satisfaction with non-invasive caries management methods.

Regarding future treatment for the same or another child, most parents (96%) across all study groups reported choosing non-invasive caries management methods (Fig. 3A). 92.5% of parents and 94% of children reported satisfaction with the treatment’s aesthetic outcome and the appearance of their teeth, respectively (Fig. 3B and C). A regression model was used to predict parental satisfaction, but it had low explanatory power (Tjur’s R^2^ = 0.10) and found no significant differences between the groups, as shown in Table 3. The absence of visible plaque was a significant factor in parental satisfaction with the aesthetic outcome (Supplementary material, Table S3). Parents reported no adverse effects beyond the outcome measures.

**Table 3:** Association between intervention groups and parental satisfaction: odds ratios and 95%CI from the adjusted regression model.

	OR^†^	95%CI^†^	*p*-value
Intervention			
P2—Placebo single applications, twice per year	–	–	
P1—Placebo one annual regime (four applications, one week apart)	1.19	0.52, 2.77	0.7
SDF1—SDF one annual regime (four applications, one week apart)	0.57	0.26, 1.22	0.2
SDF2—ADF single applications, twice per year	1.12	0.49, 2.56	0.8
TF1—Tiefenfluorid one annual regime (four applications, one week apart)	0.96	0.43, 2.15	> 0.9
TF2—Tiefenfluorid single applications, twice per year	1.65	0.71, 3.90	0.2

^†^*OR* Odds Ratio, *CI* Confidence Interval.

## Discussion

In this study, we aimed to investigate the effects of non-invasive caries management interventions based on SDF, on a set of PCOs, namely major and minor complications, and child-patient and parent satisfaction. We found the biannual SDF application significantly reduced the odds of major complications by 20% (95%CI [11%, 72%], *p* < 0.001), while the other interventions, including annual SDF, did not show a significant difference compared to the placebo. SDF-biannually also substantially reduced the likelihood of minor complications by 84% (95%CI [50%, 95%], *p* = 0.002). Almost all parents (96%) from all study groups reported choosing non-invasive caries management methods in the future. In terms of satisfaction, the effects of the different interventions were not statistically significant.

This clinical trial has limitations, such as potential performance bias due to unblinded operators and a single-institution setting, which may limit generalisability. However, the use of PCOs, including major complications (including pain, need for endodontic treatment or tooth extraction), and child and parent satisfaction, provide valuable information on the acceptability and impact of interventions. PCOs are important for assessing treatments effectiveness, relevance, and impact on patients, ensuring their appropriateness and a more satisfying experience. Additionally, the absence of stratification based on children’s prior caries experience—ranging from extensive caries lesions to minor lesions restricted to the enamel—represents another limitation, potentially influencing the uniformity of treatment effects across diverse caries histories. However, to mitigate this limitation, we performed a logistic regression analysis that identified significant predictors of treatment outcome, such as age, parental brushing habits and baseline carious lesion activity. This analysis, detailed in Supplementary Table 2, showed that despite the wide range of caries experience, the SDF2 group consistently had reduced odds of major complications, highlighting the efficacy of the intervention across different conditions. The factorial design and placebo control were implemented to minimise the influence of the Hawthorne effect by ensuring participant blinding and the use of inactive treatments. In addition, our regression analysis was adjusted for variables that could capture changes in behaviour due to awareness of being observed, allowing us to better isolate and understand the specific effects of the drug, protocol and their interactions.The raw data are available^17^ and might enable future individual patient data meta-analyses.

SDF has attracted interest as a minimally invasive approach to caries treatment, but clinical data on novel fluoride formulations, such as those containing copper, remain limited. This gap was the rationale for our study. While we observed efficacy with SDF, the evidence for the efficacy of TF was less conclusive. Further research is needed to determine whether these results are due to the composition of the TF, the application protocol (annual vs. bi-annual), or some other influencing factor. On the other hand, clinical trials evaluating the efficacy of SDF have mainly focused on technical outcomes, such as arresting caries progression and preserving tooth structure with high dentine caries arrest rates consistently found ^5,10,18^. Seifo et al.^6^ conducted an umbrella review of 11 systematic reviews and found that SDF effectively prevented and arrested coronal carious lesions in primary and permanent teeth. It outperformed fluoride varnish, glass ionomer cement, and Atraumatic Restorative Treatment for lesion arrest rate. The main side effect was the black staining of the carious lesions. Although technical outcomes are important, PCOs matters to patients and often go uninvestigated and unreported. Therefore, our study investigated the effects of non-invasive caries management interventions on outcomes such as major and minor complications, patient and parent satisfaction, and technical outcomes. We found high child-patient and parent satisfaction with SDF, which aligns with previous studies^19^. A network meta-analysis found no differences in oral health-related quality of life between SDF and other non-surgical treatments for dental caries in children^20^ Duangthip^21^ also found that using SDF for caries arrest in preschool children is safe, with no significant adverse effects reported by parents.

Preliminary results from our study also confirm the efficacy of SDF in both preventing and arresting the progression of caries in primary teeth, as shown in Supplementary Table 4 and supported by previous research^6^. More importantly, our research has revealed positive results centred on the patients themselves. These results emphasise the importance of considering the application type and protocol when using SDF for caries management. Our results suggest that the biannual application of SDF with BM, for dental caries is effective in preventing major complications at the patient level. In contrast, annual application with BM did not show a significant difference from the placebo with BM in terms of major complications. While more research is required to explore the long-term impact of SDF and other non-invasive caries management interventions on PCOs, our study further supports their use in children.

The results of our study, along with the increased interest in non-invasive treatments during the COVID-19 pandemic^22^, suggest that the use of SDF could be a valuable addition to caries management protocols. The national survey of Latvian dentists^22^ showed a general openness to incorporating non-invasive treatments, such as SDF. Nevertheless, there is a need to find the best way to get the uptake of these treatments by dentists, which might include better dissemination of information, hands-on training, and reliable evidence to support their use and offer them to parents. The findings suggest that parents and child patients are receptive to non-invasive options and may not be the barrier to their use, as is sometimes assumed by dentists. It is important that options are presented for parents and dentists, together with child patients, can make the best decision for the child, with them at the centre of the treatment planning.

In conclusion, our findings suggest that the use of SDF-biannually together with BM effectively prevents PCOs as major and minimal complications and parents and children are highly satisfied with the non-invasive treatments. This clinical trial adds to the growing evidence supporting using non-invasive caries management interventions for children. There was a combined approach to managing the lesions where behaviour change was promoted with the parent in a supportive manner. This is a recommended part of this approach to caries management^23^. The combined use of the medicament and behaviour change makes it difficult to ascertain the individual effect of each on the results. Further research could evaluate the long-term effectiveness of non-invasive treatments and the impact of training on the adoption and effectiveness of non-invasive treatments in clinical practice.

## Methods

### Ethics and protocol registration

The study protocol was approved by the Riga Stradins University Ethics Committee (Nr. 6-1/06/20) on 28/05/2020. The study strictly adhered to the guidelines outlined in the Declaration of Helsinki^24^ with the parents or legal guardians of all participants who provided their written informed consent. Subsequently, the study commenced, and the protocol in English was published, on 30/06/2021 in the ISRCTN registry (ISRCTN17005348, https://www.isrctn.com/ISRCTN17005348). The ethics committee amended and approved the protocol to include children with genetic disorders, autism, and heart disease (2-PĒK-4/627/2022). Participants were between September 2020 and May 2021, with follow-up examinations conducted from September 2021 to August 2022. Participants were allowed to participate voluntarily and were guaranteed conventional treatment if they declined. It was an additional treatment option during the waiting period for conventional treatment, addressing the limited availability of public services for children in Latvia. The study followed standard emergency intervention protocols from the Institute of Stomatology of Riga Stradins University (IS-RSU). Riva Star SDF and Tiefenfluorid were the two medications used in the study for caries treatment. Both are commercially available and are registered for professional use in Latvia. Riva Star SDF contains 35–40% silver fluoride and 15–20% ammonium^25^, while Tiefenfluorid contains 0.4% CuSiF_6_, 10.9% MgSiF_6_, 0.1% NaF, and 9.6% Ca(OH)_2_^25^. This clinical trial is reported according to the CONSORT-Outcomes (for combined completion of CONSORT 2010^26^ and CONSORT-Outcomes 2022 items^27^ (Supplementary Table S1) involved six randomised groups.

### Study design and setting

The superiority randomised single-blind clinical trial with six parallel arms was conducted at the IS-RSU Paediatric Department in Riga, Latvia, from 01/09/2020 to 01/08/2022.

### Sample size calculation

To achieve a study power of 80% and an alpha error of 0.05, we calculated the sample size for a three-treatment factorial design with two protocols, requiring 49 children for each group to detect a 20% difference^28^ in major complication proportions^29^. To account for possible losses during the observation time, 294 children were needed. To ensure sufficient enrollment, we planned to involve 70 children in each group, resulting in 420 children across all groups. Our sample size calculation was based on which caries treatment method would show a lower frequency of complications in managing early childhood caries compared with placebo. The allocation ratio was set to 1:1

### Participant selection

Parents were invited to participate through social media and consultations at IS-RSU. The inclusion criteria were: children up to 6 years of age with at least one ICDAS-3 lesion and whose parents provided informed consent. The exclusion criteria were: children who had received previously SDF treatment, refusal of masking or randomisation.

Eligible participants were referred to a clinical operator (IM) at the IS-RSU, who provided information on the study, treatment groups, and potential adverse effects. Informed consent was obtained from all the parents.

### Clinical examination

Clinical examinations were conducted by a trained and calibrated examiner (IM, Intra-examiner kappa: 0.853 for ICDAS 1 level; non-cavitated enamel lesion level; k = 0.872 for activity) who obtained the child’s history and used a pre-existing questionnaire to collect information on health, dietary habits, hygiene practices, and previous dental experiences. The presence or absence of visible plaque was recorded, and carious lesions were assessed using ICDAS^30^ codes, with simplified registration of filled surfaces. Lesion activity was assessed according to the Nyvad criteria^31^ and complications were registered as PUFA code^30^. Carious lesions were registered at tooth level, choosing the highest score of surface/surfaces. Parents received tailored advice on caries prevention based on the child’s needs.

The data about oral hygiene habits and nutrition were collected using motivational interviewing. The researcher (IM) was trained in two theoretical sessions and one practical activity on motivational interviewing before the beginning of the study. After gaining information from parents, data were entered in an *ad-hoc* form.

### Randomisation: sequence generation, allocation concealment and implementation

The randomisation process was managed by an investigator (IM) using a computer-generated simple randomisation sequence with a 1:1 allocation ratio. To enhance allocation concealment and to address concerns about potential bias, coloured cards were introduced. Each card, coded with a colour on one side for group identification, was presented with the white side facing up to ensure that allocation remained concealed until the moment of allocation. This procedure was designed to maintain the randomness and integrity of the allocation process despite the logistical challenges and space and resource constraints presented by the COVID-19 pandemic.

### Interventions

The intervention was multi-component according to the Medical Research Council definition^32^, and consisted of two parts:

Component (1) All participants’ parents received a behavioural modification (BM) intervention using passive interviewing to collect data whilst raising awareness of the importance of good oral hygiene and dietary practices and the use of motivational interviewing principles to help parents focus on changing one or two habits, emphasising toothpaste containing more than 1000 ppm fluoride. Counseling sessions, lasting 45 min or more, were provided individually to each child’s family.

Component (2) Carious lesions were treated with one of three compounds: placebo (P) (H_2_O), Tiefenfluorid (TF) (0.4% CuSiF_6_ × 6 H_2_O, 10.9% MgSiF_6_ × 6 H_2_O, 0.1% NaF, 9.6% Ca(OH)_2_) from Humanchemie GmbH, Alfeld (Leine), Germany, or SDF (35–40% silver fluoride, 15–20% ammonium) from SDI Riva Star (SDI Bayswater, Victoria, Australia). Water was selected as the placebo to provide a neutral baseline^33^. The SDF was acquired commercially. TF was donated by the company, that had no involvement in the study’s design, analysis, or dissemination. We did not receive a certificate of analysis from the manufacturer, nor did we conduct an independent verification of the contents.

There were two application regimes for each of the compounds (1) involving four weekly baseline applications, while the biannual protocol (2) entailed applications at six-month intervals.).

This meant six multi-component intervention protocols were as follows:

1. P1: Placebo used with one annual regime (four applications, one week apart) with behavioural modification;
2. P2: Placebo used with single applications twice per year and behavioural modification at both visits;
3. TF1: Tiefenfluoride used with one annual regime (four applications, one week apart) with behavioural modification;
4. TF2: Tiefenfluorid used with a single application twice per year and behavioural modification at both visits;
5. SDF1: SDF used with one annual regime (four applications, one week apart) with behavioural modification;
6. SDF2: SDF used with single applications twice per year and behavioural modification at both visits.

Each child was treated individually, and the interventions were carried out by one of the six paediatric dentists at the clinic, who were trained in advance on the study protocol and the application procedures for all the products used in the study.

### Operator and participant blinding

Several measures were taken to blind parents and children to the arm in which they were allocated:

1. To reduce the impact of taste, we used standardised communication during the application process and informed the participants in advance about the possibility of a sweet or unusual taste. In cases where taste sensitivity was expected, we used a cotton roll with a sweet toothpaste to isolate the lesion and minimise taste perception.
2. All parents were informed about possible delayed black pigmentation from the substance applied and that the arrested lesions may become dark over time.

### Baseline characteristics

At baseline, demographic information was collected, and parents provided information about toothbrushing frequency, toothpaste use, and the consumption of sweets and sugary drinks. The researcher (IM) conducted a clinical examination to record the presence of visible plaque, d1mft and d3mft indices, and the number of active lesions.

### Follow-up examination

Patients who received biannual interventions were recalled at 6 and 12 months, while patients who received annual interventions were recalled for evaluation only 12 months after the intervention. Two investigators conducted follow-up examinations. This approach was used to further validate our findings, with the second examiner demonstrating an inter-examiner kappa of 0.80 and an intra-examiner kappa of 0.70, ensuring that our assessment process was robust and consistent. No interim analyses or stopping guidelines were implemented in this study. Before the follow-up examinations, both examiners received additional training on motivational interviewing (two theoretical classes and one practical activity) to collect data about changes in behaviour related to oral hygiene and nutrition during the interview. No interim analyses or stopping guidelines were implemented in this study.

### Outcomes and outcome measures

#### Primary Outcome (major complications)

The effectiveness of the intervention was defined as the absence of major complications. Major complications were defined as dental pain, emergency visits, tooth extraction, or endodontic treatment within the last 12 months. The assessment was through a) questioning the parent about any dental emergencies or complaints over the past year and, b) from the patient’s records about visits, emergencies, and general anaesthesia sessions. and c) from clinical examination using the same methodology as in the baseline examination for healthy, filled, carious, and missing teeth and whether there was evidence of pulpal or periodontal pathology using the PUFA code^30^. For our analysis, we measured outcomes on a per-patient basis. Patients with any complications were categorised as cases and those without were categorised as successes.

#### Secondary Outcomes

1. Minor complications—composite outcome comprising lesion activity measured in Nyvad criteria^31^ or lesion progression in the 12-month follow-up defined as a positive change in ICDAS baseline status, any new lesion or ongoing activity/progression of previously treated lesions
2. Satisfaction:
  - Parent would be happy to receive that treatment again for the same child or another of their children (5-point Likert scale);
  - Parental satisfaction with the aesthetic result (5-point Likert scale); and
  - Child’s satisfaction with the aesthetic result (3-point visual analogue scale).

By employing these composite variables, we aimed to capture important treatment outcomes for parents and children and ensure that our findings were patient-centred. Any adverse effects reported by parents regarding the impact of the treatment on their children were recorded. No changes were made to the trial outcomes after the trial was commenced.

#### Covariates

1. Sex (female, male) and age (in months) of the participants.
2. Presence of visible plaque (yes or no). Plaque presence was visually assessed using an examination light, and no probe was utilised prior to the ICDAS measurement.
3. Frequency of tooth brushing (twice or more times per day; every evening; in mornings or less than once per day; seldom or never) and how often parents brush their children’s teeth (every evening; frequent if more than three times per week; seldom or never).
4. Intervention adherence was evaluated to better understand its impact on the outcome. Adherence was optimal if the participant received all intervention sessions as per the group-according protocol, was less frequent if the participant missed at least one planned visit, and was more frequent if the participant came to additional visits during the follow-up period.
5. Cooperation was assessed using a modified Venham^34^ scale and categorised as good (Venham’s codes 0–1), average (codes 2–3), or low (codes 4–5).
6. Number of active lesions at baseline as a covariate.

These variables were included to control for their potential influence on the outcome and isolate the intervention’s effect.

### Statistical analysis

The data were analysed on an intention-to-treat basis, with the child as the unit of analysis. Clinical records data were analysed in R after being entered into a secure online form with pseudonymised patient codes. The analysis population for our study was defined as participants who received at least one intervention and had at least one follow-up assessment. Participants who dropped out of the study or were lost to follow-up were excluded from the analysis population. The data were cleaned and verified against clinical records, and missing or unverifiable data were removed. The outcome analysis population was defined as the intention-to-treat population, whereby all randomised participants were included in the analysis regardless of their adherence to the trial protocol. The pattern of missing data was evaluated using MCAR from the Naniar R package. An exploratory analysis was performed to generate summary tables and visualisations. Proportion tests were used to compare the incidence of major and minor complications across the intervention groups in the clinical trial, allowing for a quantitative assessment of the impact of each intervention and identifying significant differences (*p* < 0.05) in their effectiveness. We used a GLM model to examine the association between various formulations/protocols and major complications, minor complications, and patient satisfaction while adjusting for covariates. The patient was the unit of analysis, and we accounted for clustering effects by fitting a random intercept model for each child. None of the outcome data were excluded from the analysis. Results were reported as odds ratios (OR) with 95% confidence intervals (95%CI) and a *p*-value as a measure of statistical significance.

### Ethics approval

The study protocol was approved by the Riga Stradins University Ethics Committee (Nr. 6-1/06/20). During the study, we updated the protocol, changed inclusion criteria, and obtained new approval from the Ethics Committee before the publication of the results (2-PĒK-4/627/2022). The study obtained written informed consent from parents before enrolling their children. The consent form contained the study’s nature, objective, procedures, risks, benefits, and confidentiality measures. The parents were given enough time to review and ask questions before signing the form. Participation was voluntary, and patients could withdraw without prejudice. Informed consent forms are securely stored, with limited access only, with the permission of the principal investigator to ensure confidentiality and ethical compliance. The research strictly adhered to the guidelines outlined in the Declaration of Helsinki^24^.

### Supplementary Information

Supplementary Information.

## Data Availability

The datasets generated and/or analysed during the current study are available in the Zenodo repository: https://zenodo.org/record/7677435.
