# Supplementary material for: Clinical effectiveness/child-patient and parent satisfaction of two topical fluoride treatments for caries: a randomised clinical trial
Source: Sci Rep. 2024 Apr 7;14:8123. doi: 10.1038/s41598-024-58850-w (PMC10998894; doi:10.1038/s41598-024-58850-w)
Supplement: Supplementary file 1 — Supplementary Information. [file 41598_2024_58850_MOESM1_ESM.pdf]

SUPPLEMENTARY MATERIAL

S1 Supplementary Figure 1. Unadjusted factorial analysis of major and minor complications by agent (P = placebo; SDF = silver diamine fluoride; TF = deep fluoride) and frequency (Intensive annual four-week intervention with weekly applications; Biannual = applications at six-month intervals). Percentages and standard deviations are shown.

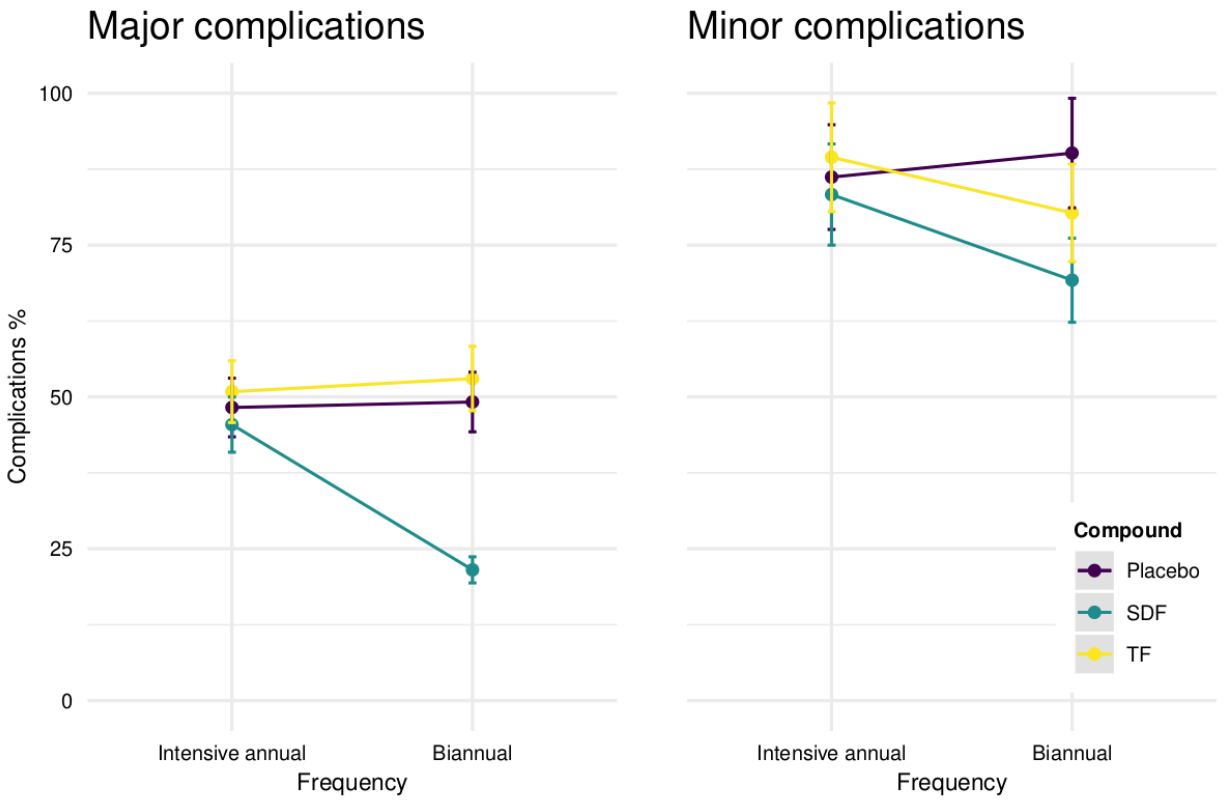

S1: Supplementary Table 1. Adherence to treatment by group.

	P1, N = 58[1]	P2, N = 61[1]	TF1, N = 57[1]	TF2, N = 66[1]	SDF1, N = 66[1]	SDF2, N = 65[1]
Optimal	48 (83%)	46 (75%)	50 (88%)	50 (76%)	52 (79%)	47 (72%)
Suboptimal	10 (17%)	15 (25%)	7 (12%)	16 (24%)	14 (21%)	18 (28%)

[1] n (%)

S2: Supplementary Table 2. Major and minor complications covariates. Adherence had a negative and significant effect for "More frequent," while rare or no tooth brushing had a negative and significant effect. Baseline active lesions had a negative and significant effect, while gender, plaque, tooth brushing frequency and cooperation were not significant. The effect of age in months was statistically significant, but the lower limit of the 95% CI suggests that the magnitude of the effect could be small or clinically insignificant.

	Major complications			Minor complications		
	**OR**†	**95% CI**†	**p-value**	**OR**†	**95% CI**†	**p-value**
**Gender**						
Female	—	—		—	—	
Male	0.90	0.55, 1.47	0.7	0.79	0.41, 1.53	0.5
**Age (months)**	1.02	1.00, 1.04	0.029	1.04	1.02, 1.07	<0.001
**Plaque**						
Not visible	—	—		—	—	
Visible	1.56	0.87, 2.79	0.1	5.27	2.22, 12.5	<0.001
**Toothbrushing frequency**						
Optimal (twice or at night)	—	—		—	—	
Suboptimal (in mornings or less than once per day)	1.21	0.65, 2.26	0.5	1.08	0.37, 3.18	0.9
**Parental brushing**						
Every day	—	—		—	—	
Frequently or sometimes	1.28	0.71, 2.30	0.4	1.67	0.70, 4.00	0.3
Rarely or never	2.14	1.03, 4.43	0.041	0.95	0.32, 2.83	>0.9
**Adherence to intervention protocol**						
Optimal	—	—		—	—	
Suboptimal	0.98	0.55, 1.77	<0.9	0.58	0.25, 1.34	0.2
**Child cooperation**						
Good	—	—		—	—	
Average	0.89	0.47, 1.69	0.7	2.04	0.90, 4.59	0.087
Low	1.64	0.90, 2.99	0.1	3.20	1.40, 7.31	0.006
**Active lesions at baseline**	1.14	1.07, 1.21	<0.001	1.11	1.02, 1.22	0.022

† OR = Odds Ratio, CI = Confidence Interval

S3: Supplementary Table 3. Parental satisfaction with the appearance of the child's teeth covariates. The satisfaction increased with the age of participants and was higher when plaque was not visible. The effects of minor complications, major complications, and pain experience on satisfaction were not statistically significant.

	**OR**†	**95% CI**†	**p-value**
**Gender**			
Female	—	—	
Male	1.06	0.66, 1.69	0.8
**Age(months)**	1.04	1.02, 1.06	<0.001
**Plaque**			
Visible	—	—	
Not visible	1.93	1.15, 3.27	0.013
**Minor complications**			
No	—	—	
Yes	0.72	0.34, 1.48	0.4
**Major complications**			
No	—	—	
Yes	1.27	0.74, 2.19	0.4
**Pain experience**			
No experience	—	—	
Pain experience	1.25	0.57, 2.87	0.6

† OR = Odds Ratio, CI = Confidence Interval

S4. Change in lesion status between treatment groups from baseline to 12-month follow-up.

Group	Active lesions at baseline	Final status		
		Active	Inactive	Restored/Missed
P1 Placebo Annual	402	175	81	146
P2 Placebo Biannual	412	169	102	141
TF1 Tiefenfluorid Annual	517	252	143	122
TF2 Tiefenfluorid Biannual	401	161	157	83
SDF1 SDF Annual	562	191	240	131
SDF2 SDF Biannual	349	109	176	64

Cochran-Mantel-Haenszel $M^2 = 111.72$, df = 2, p-value < 0.001
